# A Scoping Review of Aging Experiences Among Culturally and Linguistically Diverse People in Australia: Toward Better Aging Policy and Cultural Well-Being for Migrant and Refugee Adults

**DOI:** 10.1093/geront/gnab191

**Published:** 2021-12-30

**Authors:** Nichole Georgeou, Spyros Schismenos, Nidhi Wali, Karin Mackay, Elfa Moraitakis

**Affiliations:** School of Social Sciences, Humanitarian and Development Research Initiative (HADRI), Western Sydney University, Sydney, New South Wales, Australia; School of Social Sciences, Humanitarian and Development Research Initiative (HADRI), Western Sydney University, Sydney, New South Wales, Australia; School of Social Sciences, Humanitarian and Development Research Initiative (HADRI), Western Sydney University, Sydney, New South Wales, Australia; School of Education, Humanitarian and Development Research Initiative (HADRI), Western Sydney University, Sydney, New South Wales, Australia; SydWest Multicultural Services, Sydney, New South Wales, Australia

**Keywords:** Aged care, Ethnic group, Migrant, Refugee, Social integration

## Abstract

**Background and Objectives:**

Australia’s population is growing, aging, and becoming more ethnically diverse, resulting in barriers and challenges around social inclusion for non-English-speaking migrants and refugees. This scoping review investigates the experiences of aging within Australia among older adults from culturally and linguistically diverse (CALD) migrant and refugee backgrounds to identify barriers to social integration.

**Research Design and Methods:**

A scoping review of English language literature and gray literature on the experiences of aging among CALD migrants and refugees living in Australia was conducted from January 2000 to January 2021, according to Arksey and O’Malley’s review process. A total of 34 studies were identified for analysis.

**Results:**

Three primary themes were identified: (a) sociocultural similarities in settlement experiences, (b) engagement with technology for social connection, and (c) engagement with family and community networks.

**Discussion and Implications:**

Aging, language, sociocultural, and technology contexts shape attitudes to belonging, as well as access to sociomedical services. We argue a cultural well-being framework may assist in developing policy for improved social integration of older CALD adults. As the focus is on social and cultural experiences, all studies with a primary focus on medical and other chronic conditions were excluded. Future studies could include health-related articles to present a more comprehensive approach regarding older CALD adult needs. Follow-up research could focus on the impact of the coronavirus disease 2019 pandemic on the experiences of older adults in Australia, in particular those of CALD backgrounds.

## Australia’s Aging Population

In June 1981, Australia’s population was 14.92 million; in December 2020, it was 25.69 million, an increase of over 10 million people in 40 years. Over half the population is located in two states, New South Wales (NSW) and Victoria. One third of all older Australians live in NSW, which has a population of 8 million, of whom 5.2 million (65%) live in the capital Sydney and its surrounds. One quarter of all older Australians live in the state of Victoria, which has a population of 6.7 million, of whom 5 million live in Melbourne (74.6%; Australian Institute of Health and Welfare [AIHW], 2018).

Australian population growth comes from natural increase and migration, with the latter being more consistently significant than the former from 2006 to 2015 (Phillips & Simon-Davies, 2017). Multiculturalism as official policy dates from the 1970s onward, leading to diverse immigration and a shift in source countries. Recent migration from Asia—China, India, Philippines, Vietnam, Malaysia, and Sri Lanka—has resulted in well over 2 million older adults from culturally and linguistically diverse (CALD) backgrounds living in Australia (Australian Bureau of Statistics [ABS], 2017; AIHW, 2018). In the year ending June 30, 2020, almost 30% of the population was born overseas. Indian migrants are now the second largest group of all migrants to Australia (721,000), with the Chinese third (650,000). Of the top 10 migration source countries, Vietnam (270,000) is sixth, Malaysia (177,000) is ninth, and Sri Lanka (147,000) is 10th. The 2016 census reported that 21% of all Australian homes speak a primary language other than English. There are in fact over 300 identified languages spoken in Australian homes (ABS, 2021). In South Western Sydney, fewer than half of residents (46.3%) spoke only English at home, a figure well below the NSW state average (68.5%). In some local government areas, speaking non-English languages was even more common: Fairfield (75.5%), Canterbury-Bankstown (63.7%), and Liverpool (57.2%; NSW Government, 2019, p. 13).

Australia’s immigration policy aims to attract 160,000 migrants in 2021–2022. Just under half of these (79,600) are in the skilled stream, which includes employer-sponsored positions (22,000), global talent (15,000), and business innovation and investment (13,500). The other main group (77,300) is the family stream, which is heavily geared toward partners (72,300) rather than parents (4,500; Department of Home Affairs, Australian Government [DoHA], 2021a). People thus come to settle in Australia for work and may bring their partner, but they will grow older in Australia, and most without their parents. The same applies to the humanitarian or asylum streams, where 58% of applicants were between 25 and 44, and fewer than 20 of 950 visa determinations made in the month of June 2021 came from those aged older than 65 (DoHA, 2021b).

While the number of older CALD Australians is set to increase further, Australian adults are also living longer. Children born between 1970 and 1972 could on average expect to live until 74.5 for a female and 67.8 for a male. Three decades later, a female born in 2002–2004 could on average expect to live to the age of 83 (a gain of 8.5 years), and a male until 78.1 (a gain of 10.3 years). In 2017, around 3.8 million Australians (just under one in seven people) were aged 65 years and older. By 2057, Australia is estimated to have 8.8 million older adults (one in five people), and by 2097 this increases to one in four (12.8 million people; AIHW, 2018).

While aging presents a variety of challenges for all people, such challenges are exacerbated for older CALD adults. Many overseas-born Australians face substantial barriers to being fully integrated into the wider community due to limited English proficiency, digital literacy, small social networks, and little previous exposure to Australian society, in particular health and social services (Atwell et al., 2007; Caidi et al., 2020; Du & Xu, 2020; Ethnic Communities’ Council of Victoria [ECCV], 2012; Federation of Ethnic Communities’ Councils of Australia [FECCA], 2015; Rao et al., 2006; Walker et al., 2013). Unavoidably, such issues affect the capability of older CALD adults to access and engage with the essential support and services that contribute to good outcomes and improved quality of life (Al Abed et al., 2013; Atwell et al., 2007; Fountain et al., 2019; Johnstone & Kanitsaki, 2008; Refugee Health Research Centre, 2005).

A plethora of studies confirm that isolation—including practical, social, and emotional loneliness, depression, and stigma—is a common outcome for older CALD migrants and refugees (Atwell et al., 2007; Du & Xu, 2020; Fountain et al., 2019; Panagiotopoulos et al., 2013; Refugee Health Research Centre, 2005; Walker et al., 2013). From 2020 and the onset of the coronavirus disease 2019 (COVID-19) pandemic in Australia, these concerns amplified as public health restrictions on movement created concerns about social isolation for all people and for older adults in particular.

Most older Australians live outside of aged care facilities and do not engage with other forms of aged care support. In Australia in 2017–2018, just 1.3 million people (three out of 10 older adults) received some form of aged care (Atkins & Baldassar, 2020; Department of Health, Australian Government [DoH], 2020); the majority of these received home-based care and support, and relatively few lived in residential care (DoH, 2020). A majority (seven of every 10 older Australians) live in the community. Among older adults in more recently established CALD communities in Australia, there is extreme resistance to residential aged care. A higher acceptance of residential aged care exists among communities that have a longer history of settlement in Australia (Rees & McCallum, 2018, p. 4).

Before COVID-19, most older adults lived alone, suffered from decreased mobility, and were at risk of social isolation (Blunden et al., 2019). When combined with COVID-19 restrictions, such barriers to social isolation are exacerbated as many older adults cannot walk for an hour a day, so are reliant on compassionate care visits from others for human contact. Identifying and addressing existing barriers that prevent social inclusion among older CALD adults in Australia, as well as devising supporting strategies, will help minimize such barriers in a post-COVID-19 world.

## Method

This scoping review investigates both the traditional and gray literature on the aging experiences of older adults from CALD migrant and refugee backgrounds in Australia to identify issues in the care of older CALD adults. The analysis is based on the four-stage framework of Arksey and O’Malley (2005): (a) identifying research questions, (b) identifying studies, (c) selecting studies, and (d) collating and reporting findings. The method has been used by many scholars in gerontology (Grenier et al., 2020; Hausknecht et al., 2020) and in other disciplines (Pham et al., 2014), including in occupational health and safety (Schismenos et al., 2021) and development studies (Wali et al., 2020).

### Research Question

This scoping review explored literature on the experiences of aging among older adults from CALD migrant and refugee backgrounds and asked: *What are the specific cultural and social experiences of older CALD adults from migrant and refugee backgrounds with respect to aging in Australia?*

### Search Strategy

The following combination of search terms was used across various data sources for the identification of relevant studies:

(“elder” OR “older” OR “aging”) AND (“CALD” OR “culturally and linguistically diverse” OR “migrant” OR “refugee” OR “overseas-born”) AND (“Australia”)

### Identification of Studies

The following search engines were selected: Oxford Academic, ProQuest, Sage Journals, Science Direct, and Wiley Online. The engines cover a range of related fields (i.e., aged care, migration, CALD). In addition, 10 academic journals were selected due to their high relevance to the topic. “Gray” literature (literature developed or published by international, multinational, and national organizations and other entities whose core business is not “traditional” academic publications) was also searched from organizations selected in consultation with industry experts. Table 1 presents the list of academic journals and “gray” literature sources selected for this study.

**Table 1.** Summary of Inclusive Studies

Academic journals	Gray literature
*Ageing and Society* *Australasian Journal of Ageing* *Journal of Cross-cultural Gerontology* *Journal of International Migration and Integration* *Journal of Immigration and Minority Health* *Journal of Ethnic and Migration Studies* *Population, Space and Place* *Social Science & Medicine* *The Journals of Gerontology: Series B* *The Gerontologist*	Australian Institute of Health and Welfare Department of Health, Australian Government Ethnic Communities’ Council of Victoria Federation of Ethnic Communities’ Councils of Australia Royal Commission into Aged Care Quality and Safety Council on the Ageing New South Wales Refugee Support Network

### Inclusion and Exclusion Criteria

For the purpose of this study, only case studies, general reports (including government reports), research articles, and review articles available in English were included. The review did not exclude any study based on the design, which allowed exploring all studies including literature reviews, qualitative, quantitative, and mixed methods studies. Studies published from January 2000 to January 2021 were considered because of the launch of the multicultural policy statement “A New Agenda for Multicultural Australia” in December 1999 (Koleth, 2010). In the statement, the term “Australian multiculturalism” is included and reflects on “Australia’s diverse heritage, history, democracy, culture and identity” (National Multicultural Advisory Council, 1999, p. 5).

Excluded study types were books, book reviews, editorials and opinion pieces, studies not in English, or relevant studies where the primary focus was on dementia, fall injury, diabetes, oral health, heart failure, incontinence, cancer, or other chronic conditions, and advanced care planning. These were omitted as the primary focus of this study was on the sociocultural experiences of older CALD migrants and refugees, rather than on the health issues associated with aging.

### Selection of Studies

**Figure 1.**
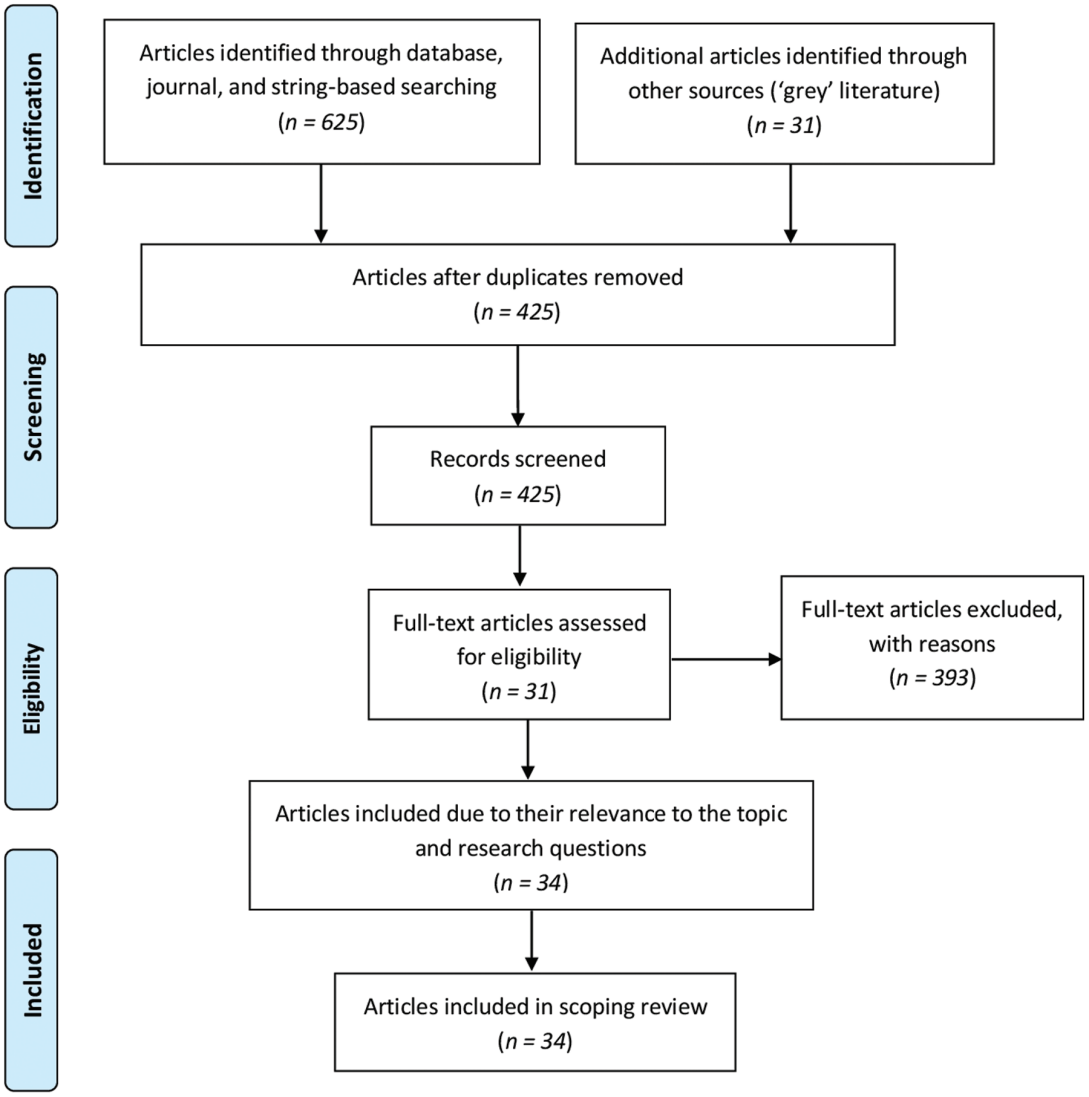
Flow chart of study selection.

The search of the five databases and 10 journals resulted in a total of 625 studies. After removing all duplicates, 394 studies remained. The advanced search options of title, abstract, and keyword screening were used (last search date: February 15, 2021). The full-text screening then resulted in 26 studies being retained. No additional articles were found through the bibliography search. The search from the gray literature resulted in 31 reports, and after full-text screening, five of these were included. Three other studies (Atwell et al., 2007; Egan & Bowes, 2013; Teshuva et al., 2019) were added due to their relevance to the topic and research questions; these were found by using the string keywords in the Google search engine. The full-text analysis thus led to a total of 34 studies for final inclusion. The entire process is visually explained in Figure 1; the Preferred Reporting Items for Systematic Reviews and Meta-Analyses guidelines were followed (Moher et al., 2010). A summary of the studies is provided in Table 2.

**Table 2.** Summary of Inclusive Studies

Study	Study design and sample	Sample characteristics	Objectives	Key findings
**Atwell et al. (2007)**	Qualitative Direct interviews with 17 community workers, and 16 service providers in Victoria	Participants were engaged with older newly arrived refugees from 14 different communities	To explore the needs of older refugees and barriers to their receiving health and aged care	Older refugees experienced changes in family dynamics, isolation, and customs that led to family conflict, stress, and mental illness Limited or no services literacy
**Caidi et al. (2020**)^a^	Qualitative Direct interviews with eight participants (average age: 67 years)	Older Chinese living in Australia (Adelaide) and Canada (Toronto)	To examine older Chinese adults’ mobility through a broader (transnational) lens	Mixed feelings about host country High dependency on family and community for daily activities Identity “dilemma” Use of social media to contact relatives/friends in origin country Interaction with local community and coethnics was beneficial
**Joffe et al. (2003)**	Qualitative Direct interviews with 100 survivors, 50 refugees, and 50 Australian/English-born persons (age: 60+ years)	Holocaust survivors, immigrant refugees, Australian/English-born persons living in Sydney	To reexamine psychosocial morbidity in an older community sample of Holocaust survivors	Holocaust survivors were in worse psychosocial condition than the other groups All groups were similar in social and instrumental functioning Severe traumas resulted in great levels of psychological morbidity
**Teshuva et al. (2019)**	Qualitative Direct interviews with 13 Jewish Holocaust survivors residing in Melbourne (age: 80+ years)	Older Jewish Holocaust survivors who used aged care community services and were born in various Eastern Europe and former Soviet Union countries	To investigate the viewpoints of older Jewish Holocaust survivors’ lived experience of using community aged care services	Need for support to maintain autonomy, have a good relationship with the carer and be understood as an individual Feelings of distress and bad memories when not treated properly by carers Need for carers to do their job well
**Teshuva and Wells (2014)**	Qualitative Direct interviews with 21 Cambodian or Jewish genocide survivors (age: 65+ years)	Survivors of the Cambodian or Jewish genocide living in Melbourne and Sydney	To investigate the aging and aged care experiences in Australia of Jewish Holocaust and older Cambodian genocide survivors	Importance of understanding older survivors’ aging and aged care experiences in the context of their entire life course and in terms of both vulnerability and resilience Trauma history could heighten survivors’ sensitivity to many aspects of the social and physical environments in residential, community, and home-based aged care settings Importance of recognizing older survivors of genocide as a distinct group of clients
**Walker et al. (2020)**	Qualitative Direct interviews with 19 family caregivers from 10 Italian and four Greek families (age: 50–91 years)	Caregivers to a family member with intellectual disability into late life	To investigate how older CALD parent caregivers experience caring for their family member with intellectual disability into late life	Identification of stressors (e.g., need to make sacrifices, physical and emotional demands, uncertain futures) Family members played a key role in providing support; however, changing values around filial responsibility were evident Need for attention to CALD families to address challenges associated with caring for their family member with intellectual disabilities
**Wilding et al. (2020)**	Qualitative Direct interviews with 51 older migrants of various ethnic backgrounds (age: 50+ years)	Older CALD adults who migrated to Australia due to ethnic violence and civil war conditions and used digital media	To draw attention to the specific role of the emotions that are circulated through digital media interactions and practices	The capacity of digital media circulated emotions and supported affective economies
**Winterton and Warburton (2012)**	Qualitative Direct interviews with 16 older migrants living in their community for at least 11 years (age: 64–98 years)	Retired migrants living in two small rural communities in the Hume region, Victoria	To investigate how rural older retirement migrants and long-term residents in Australia use place to sustain and build a sense of self at a time when many are susceptible to age-related loss	Aspects of rurality were significant to older adults’ positive identity Place identity was supported by distinctiveness, continuity, self-esteem, and self-efficacy Rural communities were beneficial in terms of individuals’ identity maintenance
**Yeboah et al. (2013)**	Qualitative Direct interviews with 20 residents of four nursing homes in Melbourne (age: 61–92)	English-speaking older CALD adults, born in Europe, Russia, and India	To investigate nursing home relocation experiences of overseas-born older CALD adults	Loss (physical, relational, or support) was the main reason for relocation to residential aged care Cultural considerations were important in assessing the quality of a nursing home and in strategies used for settling into the home
**Chou (2007)**	Qualitative Two waves of direct interviews—Wave 1: 431 participants; Wave 2: 359 participants (age: 50+ years)	Migrants of various ethnic backgrounds, living in Australia	To examine whether origin of countries and visa types predicted psychological distress over a period of 1 year, and whether their association changed after factors in health, social roles, cohort effect, and social support were adjusted	Psychological distress was persistent and related to visa classification and reason for immigration Psychological distress of “newcomers” increased over a period of 1 year Visa type affected mental health Older migrants from non-Western/developed countries reported higher psychological distress
**Fountain et al. (2019)**	Qualitative Delphi method Two rounds of interviews—Round 1: 112 participants; Round 2: 58 participants who participated in Round 1	Australian-based relative stakeholders including older adults, carers, government agencies and departments, community services/organizations, and aged care facilities	To identify research priorities related to older adults and natural hazards in Australia	Need for every relative stakeholder to be engaged in disaster management Lack of disaster education in older migrants increased risk exposures and affected response Social isolation and stigmatization could affect disaster response capabilities Need for further research
**Egan and Bowes (2013)**	Qualitative case study Direct interviews with five families, two prominent community members, and observations	Bhutanese community in Sydney	To (a) identify how the Bhutanese community cares for its seniors, (b) report on how this is working and what could be improved, (c) identify continuing needs within the community, and (d) address lessons for the wider Australian community	Mainstream care was not commonly accepted Language and location barriers Joined activities/events brought community members closer older adults were respected for their cultural knowledge and taking care of grandchildren
**Zannettino et al. (2015)**	Qualitative case study Direct interview with an individual	A man in his 50s described the past financial abuse of his mother by his sister	To investigate themes or factors that may be shared by older CALD adults when experiencing and responding to financial abuse	Social isolation, language barriers, and high dependence on a family member resulted in financial and emotional abuse Cultural background and ethnicity played a role in financial and emotional abuse Connections with the wider community including more relatives could protect against financial and emotional abuse
**Du and Xu (2020)**	Qualitative Focus groups with 13 late-life Chinese migrants, and direct interviews with nine other older Chinese migrants (age: 61–75 years)	Older Chinese migrants living in Adelaide, South Australia under the family reunion scheme	To advance the understanding of older migrants’ information behavior in transition and their resilience in leveraging information and technology to achieve social integration into a new country	Disconnectedness from previous information practices Perceptions of marginalization and having limited access to resources in English Language barriers Obtaining information by participating in ethnic associations, relatives, friends, and using social media for communication
**ECCV (2012)** ^a^	Qualitative Focus group with representatives of communities, older CALD adults, international students	Participants represented groups with aged care, health, transport, and settlement needs	To provide its members with the opportunity to discuss any experiences with Australian Government services and to suggest ways to improve them	Low digital and service literacy Language barriers Culturally competent communication reduced barriers
**Goodall et al. (2010)**	Qualitative Focus groups with 19 Greek and 24 Italian participants (age: 55+ years)	First generation of migrants who came to South Australia in the 1950s and 1960s	To analyze the views of older migrants living in South Australia regarding their information sources, use, barriers, and enablers to future use of information, communication and technology for accessing health information	Limited use of information, communication, technology to access daily information, and limited interest in learning how to use it Access to needed information was from multiple sources by various means such as electronic and print media
**Hugman et al. (2004)**	Qualitative Focus groups with 29 older refugees (per group), one focus group with eight community workers, and direct interviews with 10 community workers	Older refugees of various ethnic backgrounds living in Sydney, and community workers of the refugees’ communities	To explore perceptions of the issues facing older refugees, from the perspective of older refugees themselves and from workers in their communities who assist them	The impact of being a refugee was seen in the effects of trauma and of forced migration Older refugees had a sense of “aging in the wrong place” Reliance on relatives for survival Changes in family dynamics and customs
**Nau et al. (2019)**	Qualitative Focus groups with 42 older adults (mean age: 76), and direct interviews with 30 community service providers	Providers and older adults in structured physical activity programs for older adults in Victoria	To examine the perceptions of providers about strategies to improve adherence to organized physical activity among socially disadvantaged older adults	Adherence barriers included deteriorating health, lack of “belonging” and loss of motivation A range of strategies could mitigate risks to adherence and support continued participation
**Li and Southcott (2012)**	Qualitative case study Focus group, and direct interviews with the founder of Kingston Chinese Senior Citizens and eight members of the Singing Group (age: 59–85 years)	Older Chinese-origin women arrived in Australia 10 years ago either as refugees or for joining family and currently living in Victoria	To investigate the benefits in community singing groups through the experiences of older Chinese Australians	Four broad themes were identified: emotional well-being, connections with the past, shared interests, and mental and physical well-being
**O’Callaghan and Quine (2007)**	Qualitative Focus groups and direct interviews with 20 Vietnamese women living in Sydney (age: 55–75 years)	Vietnamese women experiencing difficulties in managing their medicines	To investigate the use of medicine by older migrants and to describe how women’s limited health literacy impacts on how they manage their medications	Participants’ health literacy influenced their medication use and explain the cultural reasons behind their decision to vary medicine use Concern that health professionals do not favor combining western with traditional medicines Cultural gap with doctors due to doctors’ lack of knowledge or disapproval of traditional medicine
**Walker et al. (2013**)	Qualitative Focus groups with 63 participants, and direct interviews with 27 participants (age: 69–90 years)	Greek-speaking older adults having migrated to Adelaide and Darwin either from Greece or other countries	To investigate the perceptions of older Greek-Australians toward changes in the nature of family support	“Cultures of care” remained in Greek-Australian families; however, the means for a family to assist had shifted Feelings of loneliness and fear because participants lived alone and could not fully understand English Need to better involve older migrants and their families in decisions about their care needs Need for service providers to adopt the use of new technologies to communicate with increasingly time-pressured family members
**Millard et al. (2018)**	Mixed methods Interviews, observations, and social network mapping with 15 older migrants, members of an “internet cafe”	Older migrants living in Perth, Western Australia	To understand the increasingly important role of digital citizenship in supporting the well-being of older migrants	Digital literacy could enhance older adults’ maintenance of support networks, social engagement, and their access to health care services Digital literacy support was more effective when developed through social learning systems
**Panayiotou et al. (2020)**	Mixed methods Focus group, survey, and an appraisal of translation applications with 12 older adults of Greek and Chinese origin, and 17 health care workers	Older CALD adults with no significant visual or auditory impairment and health care workers from hospitals in Melbourne	To understand the attitudes and perceptions of older adults with limited English proficiency and health care workers to use mobile translation technology for overcoming language barriers in the health care setting	Translation technology could reduce communication barriers such as accuracy of translation, possible technological learning curves, risk of mistranslation in high-risk conversation, and inability to check the accuracy of translation
**Johnstone and Kanitsaki (2008)**	Review of relevant literature	Older CALD adults living in Australia	To suggest that cultural racism is another cause of the ethnic aged disparities in health and social care in Australia	Major health and social care disparities due to educational, linguistic, cultural, geographical, social, and economic factors Cultural racism could be a cause of ethnic aged disparities and disadvantages in health and social care
**Rao et al. (2006)**	Review of relevant literature	—	To investigate the evidence related to the health and social needs and existing support systems for older CALD Australians	Heterogeneity both between and within older CALD adults Health and social needs may be particularly acute as a result of cultural and language barriers, geographical location, and the circumstances of migration
**van Gaans and Dent (2018**)^a^	Review of relevant literature (1999–2016)	—	To investigate the difficulties older Australians face when accessing health care services	Availability, accessibility, accommodation, affordability, and acceptability issues for CALD and rural populations Need to improve health care services
**Ahmad and Khan (2015)**	Qualitative Review of relevant academic and gray literature Consultation with 25 industry organizations/persons	—	To investigate whether aged care in Australia meets the needs of Muslim older adults with a special reference to South Australia	Only partial recognition of role and significance of religion in aged care Lack of services for religious/cultural practices Negative stereotypes toward residential care
**FECCA (2015)** ^a^	Review of relevant literature (2003–2014) and (1970–2013) Consultation with 37 organizations	—	To identify existing research evidence about older CALD Australians and to identify gaps in the research	Well-being and health were affected by social status Cultural transitions could cause misunderstandings or service barriers Health and well-being were affected by the length of living in Australia Lack of knowledge/understanding of available services Reliance on others for survival; however, not all migrant families “took after their own”
**Refugee Health Research Centre (2005)**	Qualitative Review of relevant literature and information from various databases Interviews with service providers and community workers	—	To (a) review the literature on refugee seniors, (b) profile seniors from key refugee communities in Victoria, (c) review of aged care services, (d) identify innovative models for addressing the needs of refugee seniors	Social isolation, loss of independence, and mental illness Past experiences, language barriers, age, length of living in Australia, and resource availability within the community increased challenges
**Al Abed et al. (2013**)^a^	Systematic review of relevant academic and gray literature (January 1990–October 2012)	—	To investigate the health care needs of older Arab-Australians and their sociocultural characteristics	Racial stereotyping affected health-seeking behaviors and health care treatment The understanding of specific cultural attributes could improve health Need for effective ways of communication to provide more culturally competent care and better health
**Panagiotopoulos et al. (2013)**	Quantitative Questionnaire with 61 British-born, and 60 Greek-born participants (age: 57+ years)	Two groups of older migrant widows in South Australia	To examine the well-being of older migrant widows in South Australia	Greek-born widows displayed higher levels of mourning rituals, worse self-rated health, and increased symptoms of depression and loneliness Both groups perceived high levels of familial social support
**Dowling et al. (2019**)^a^	Quantitative Examination of the first three waves of data from the “Building A New Life In Australia” longitudinal survey of adult humanitarian refugees living in Australia (October 2013–March 2016)	2,399 humanitarian refugees from 35 countries resettled in Australia	To examine the association between migration factors and self-rated general health of adult humanitarian refugees living in Australia	Poor general health persisted throughout the 3-year follow-up Female gender, increasing age, and postmigration financial stressors were positively associated with poorer general health Having a university degree and absence of chronic health conditions were seemingly protective against declining general health
**Goh et al. (2010)**	Qualitative Cross-sectoral survey with Chinese-origin patients, family carers, and staff	Patients, family carers, and staff from 13 mainstream and 3 Chinese-specific nursing homes	To examine the levels and rates of depression in Chinese residents living in ethno-specific nursing homes, and Chinese residents living in mainstream nursing homes in Sydney	No significant differences in resident depression levels or rates between the facility types Chinese-specific nursing homes residents had lower prescription levels of antipsychotics, possibly due to limited language barriers Impairment issues could affect socializing with other patients
**NSWRSN (2017)**	Online survey with 27 workers	11 organizations working with newly arrived refugees in New South Wales	To describe some of the practical and policy challenges	Need for inclusion of the unique and specific needs of older refugees in health, aged care, and settlement support Lack of fast-track health services to newly arrived older refugees

*Note:* CALD = culturally and linguistically diverse.

^a^We present findings that are most relevant to older migrants and refugees based in Australia from the period 2000 to 2021.

### Data Analysis

The final studies included were coded following the six-step process as detailed by Braun and Clarke (2006), including (a) familiarization with content by reading and re-reading the studies, (b) generating initial codes, (c) grouping codes into themes, (d) reviewing themes to develop a thematic map, (e) defining and naming themes, and (f) narrating themes. The initial coding process was undertaken by the corresponding author (S. Schismenos). Initial coding was conducted by the second author (S. Schismenos). The code tree and initial themes were then discussed with the third author (N. Wali). Revised themes and subthemes were then reviewed and discussed with the first author (N. Georgeou) for logic and consistency. The identified themes were finalized in consultation with an industry expert (E. Moraitakis) working with older CALD populations in the Western Sydney region, NSW. This method allowed synthesizing the large body of studies shortlisted for inclusion in the review. As the majority of studies were qualitative (24 of the 34 included studies), this method allowed an in-depth analysis and developed common themes emerging from the literature. Findings from quantitative and mixed methods complemented the qualitative data findings.

## Results

The final review included 34 studies that were a combination of academic papers and reports. The majority of studies (24 studies) were qualitative, and the remaining were a combination of quantitative, mixed methods, and reviews. Methods employed in the majority of the qualitative studies were direct interviews and focus group discussions. A theme map is available in Supplementary Materials.

The scoping review of the experiences of older CALD migrants and refugees led to three major findings:

- 1. There were similar settlement experiences and needs despite differences in culture, language, and religion.
- 2. They exhibit differing levels of agency when interacting with technology for social connection and to access sociomedical services.
- 3. Engagement with family and community networks is crucial for a sense of belonging.

Our findings suggest that older adults from CALD migrant and refugee backgrounds are a diverse and growing group within Australian society that has broadly similar needs and experiences of aging in Australia. Older CALD adults have similar needs in terms of meaningful social participation and belonging and these become even sharper when aging, especially if living alone. Factors such as immigration pathway to Australia, length of living in Australia, and level of English language proficiency affect an individual’s sense of investment in the wider community. Individual factors that affect experiences of aging include age at migration, level of education, gender, religion, income, socioeconomic status, country of origin, and geographic location of residence. The most critical barriers to a sense of belonging are language proficiency in English, services literary, and a perception that their sociocultural difference from the wider population is not valued. Limited English language proficiency or digital literacy affects older adults’ overall experience of understanding and accessing various services, limits access to information and communication, and increases dependence on relatives. Cultural differences between Australia and a person’s country of origin affect older CALD adult interactions with local communities, as well as their relationships with family members (e.g., grandchildren who were born and raised in Australia and who may not speak the “mother tongue”).

### Sociocultural Similarities in Settlement Experiences

There were three primary reasons for migrating: (a) family reunification—particularly among Chinese older adults who migrated willingly (Caidi et al., 2020; Du & Xu, 2020), (b) finding better working and living conditions, and (c) surviving (i.e., holocaust or war conditions in origin countries). The latter reasons were more common among refugees or asylum seekers, where migration is usually forced (Atwell et al., 2007; NSW Refugee Support Network, 2017).

Since the early 1970s Australia has officially adopted policies of multiculturalism and nondiscriminatory migration (Jupp, 1995), so a diversity in demographic characteristics was an expected finding. Most studies included participants’ gender and age. Three studies focused only on women—community singing groups (Li & Southcott, 2012), perceptions of Western and traditional medicine (O’Callaghan & Quine, 2007), and widowhood (Panagiotopoulos et al., 2013). Regarding participant ethnicity, most older adults were primarily from Arabic, Bhutanese, Bosnian, British, Burmese, Cambodian, Chinese, Greek, Italian, Somalian, Sri Lankan, Sudanese, and Vietnamese backgrounds. Christianity, Judaism, and Islam were identified as the main religions of most participants. The level of satisfaction for cultural practices and religious values affected, to some degree, positive experiences of aging in Australia (Ahmad & Khan, 2015; Al Abed et al., 2013). Education among older adults varied from non-/limited to skilled, depending on country of origin, age of arrival in Australia, and reasons for migrating, and was one factor affecting health and socioeconomic status (Dowling et al., 2019; FECCA, 2015; Johnstone & Kanitsaki, 2008; Rao et al., 2006). Table 2 provides further information on the details of the older adult population.

Some older CALD adults did not adjust easily to Australia’s sociocultural conditions when they first arrived or feared loss of cultural identity, values, roles, and traditions over time. Atwell et al. (2007) noted some newly arrived male refugees were concerned that they would lose their patriarchal power and respect, particularly over “their” women, because women in Australia have more rights and freedom. Du and Xu (2020) highlighted that some older Chinese parents did not feel like leading figures in Australia because they could not support their family as much as in their country of origin due to barriers with language and limited networking or interaction with the local community. Such phenomena were more common among newly arrived older adults, and Du and Xu (2020) highlighted that some migrants and refugees felt unprepared for life in Australia, or that Australia was very different from what they had initially expected.

Despite similarities in reasons for migrating, older CALD adult cohorts demonstrated significant differences to each other, especially after a period of staying. This was due to different sociocultural makeups, capabilities, such as financial status, and needs, which affected the agency. For example, Panagiotopoulos et al. (2013) presented important differences between Greek and British widows in Australia around loss of loved ones, health, “moving on,” and dependence on relatives.

Another difference was observed in family relations, as well as in perceptions regarding mainstream approaches to care. Egan and Bowes (2013) noted nursing homes were not a common solution for Bhutanese older adults who expected their family to provide support and care. Greek older adults had a similar perception, but were more willing to adjust to the “Australian way” so they would not become a burden to their relatives. Walker et al. (2013) reported that Greek older adults preferred living alone and required no formal aged care services. The study also reported that they would accept, even if they disagreed with, the idea of living in a nursing home in order to not affect their children’s family and happiness when they could no longer live independently. Yeboah et al. (2013) explained that relational, physical, and support losses were common reasons for older CALD adults to relocate to residential aged care.

Specialized infrastructures, experienced staff, and satisfactory service levels were not available in rural and regional areas of Australia (van Gaans & Dent, 2018; Winterton & Warburton, 2012), which was also characterized by a lack of services, and some inappropriate facilities for different cultural and religious groups (Ahmad & Khan, 2015; Al Abed et al., 2013). A study of Holocaust survivors’ experience of aged care services by Teshuva and Wells (2014) raised concerns over some space arrangements in aged care facilities that trigger negative memories (e.g., locked doors and windows, enclosed space, and long corridors), while Teshuva et al. (2019, p. 242) described how “the negative impacts of not being treated in ways consistent with person-centred care principles are exacerbated for trauma survivors.”

Older adults who acted as primary carers for family members with intellectual disabilities were concerned by the available services for both aging and caring matters, for example, what will happen when they grow too old and cannot take care of others, or what accommodation options are available for members with intellectual disabilities (Walker et al., 2020).

### Engagement With Technology for Social Connection

Many older CALD migrants and refugees experienced issues when interacting with technology for various purposes including communication and health and social services. Goodall et al. (2010) described the reluctance of Greek and Italian older adults who migrated in the 1950s and 1960s to use personal computers, smartphones, and tablets to receive information. Those who spoke English well preferred calling the service providers themselves or being informed by various news sources such as radio and television. Many older adults who were willing to use such digital services reported other issues. For example, there was wide dissatisfaction with phone access services, primarily due to language barriers, either when older adults could not speak or understand English sufficiently, or when there were no interpreting services available. Older adults also hesitated to interact with automated messages, while major disablers for using digital services included hearing and visual problems, limited internet access, and processes that were time-consuming. Similar findings were observed among older CALD adults who used translation apps in health care. Panayiotou et al. (2020) noted poor translation services for lesser-known languages and dialects.

The use of devices with access to social media was more common among older adults who had migrated recently from countries with higher digital literacy such as China. Caidi et al. (2020) and Du and Xu (2020) pointed out that Chinese older adults used social media to keep in touch with their relatives and friends in Australia and overseas. Wilding et al. (2020) described similar findings for Burmese, Sri Lankan, and Somalian older adults. This was possibly due to the fact that social media is a reliable, fast, and inexpensive way to stay in contact with distant people. Millard et al. (2018) stated that in addition to keeping in touch with loved ones, some adults were willing to learn how to use internet devices as this allowed them to become more independent. Some findings indicated that the use of social media, and generally sufficient digital literacy, was a critical factor for reducing loneliness, depression, and reliance on family members for survival (Caidi et al., 2020; Du & Xu, 2020; Millard et al., 2018; Wilding et al., 2020).

On the other hand, the overuse of social media and frequent communication with relatives and friends in the origin country increased the feeling of homesickness and social isolation (Caidi et al., 2020). Importantly, loneliness and social isolation were highlighted in several studies and were often linked with limited English proficiency and barriers to socializing, due to restricted networks and limited opportunities to create new connections in a new environment (Panagiotopoulos et al., 2013; Walker et al., 2013; Wilding et al., 2020).

With respect to health care, aged care, and social services in Australia, many older migrants and refugees complained that they found these complicated to navigate or unreachable; even those with a strong understanding of technology encountered major issues with language when attempting to access online health services. Most findings suggested that language barriers were major disablers and increased services’ inaccessibility (Atwell et al., 2007; Caidi et al., 2020; Du & Xu, 2020; ECCV, 2012; FECCA, 2015; Rao et al., 2006; Walker et al., 2013). Older CALD adults who did not speak English to a sufficiently fluent level did not communicate well and could not access or navigate services well in Australia.

### Engagement With Family and Community Networks

The most pertinent factors that prevented older adults from social engagement were (a) language barriers (Atwell et al., 2007; Du & Xu, 2020), (b) disabilities (Goh et al., 2010; Nau et al., 2019), and (c) traumatic experiences (Atwell et al., 2007; Joffe et al., 2003). Fountain et al. (2019) pointed out how these factors could also affect evacuation processes (e.g., a migrant may not understand that an evacuation is immediate due to flooding because of language barriers). Atwell et al. (2007) and Chou (2007) stated that preexisting mental health issues and psychological distress (e.g., due to forced migration or limited labor skills) also affected well-being and could lead to social isolation and loneliness.

According to Teshuva et al. (2019), the most encouraging enablers for engagement were the understanding of the needs of older adults as individuals and not as a homogenous group. The feeling of being listened to, familiarity, usefulness, and respect in all interactions, not only with family and community members but also with health workers, were noted as integral to feelings of being valued.

Findings indicated that older adults felt better when engaged with family members or coethnics as there were no language and other such barriers (Caidi et al., 2020; Du & Xu, 2020; FECCA, 2015; Hugman et al., 2004; Panagiotopoulos et al., 2013; Wilding et al., 2020). Du and Xu (2020), Li and Southcott (2012), Nau et al. (2019), and Winterton and Warburton (2012) pointed out that older adults who engaged with both coethnics and non-coethnics presented an increased sense of belonging and acceptance (e.g., when they participated in music and physical activities). This feeling was also observed among older migrants who lived in rural communities where local societies are smaller (Winterton & Warburton, 2012). However, some older CALD adults were vulnerable to financial and emotional abuse by their family members and people they trusted (Zannettino et al., 2015).

## Discussion

The scoping review aimed to synthesize existing studies of the cultural and social experiences of older adults from CALD migrant and refugee backgrounds in Australia. The literature highlights how aging interacts with the complex relationship between language, sociocultural, and technology contexts to shape attitudes about belonging, as well as access to sociomedical services. In this section, we first discuss the main themes that emerged from the review before suggesting that a framework of cultural well-being would be a useful approach for policymakers to reduce instances of social isolation among older CALD adults due to its holistic focus. We then explain what a cultural well-being framework would entail, and how it could be used to understand and develop policy and best practices for issues of aging, language, sociocultural context, technology, and engagement for CALD migrant and refugee cohorts in Australia. Finally, while the geographical focus of this study was Australia, literature from the United States and Canada points to similar findings, and we suggest a wider application of the cultural well-being framework in aged care service policy and provision might be beneficial.

### Reciprocal Relational Networks and Connections

Social engagement with society, local community, family, and friends is an effective solution against loneliness as it increases a sense of belonging (Tymoszuk et al., 2019). Egan and Bowes (2013) described the conditions in the Bhutanese community in Western Sydney, where older adults engaged with coethnics and contributed to community and family activities. This finding points to the centrality of the expression of culture through reciprocal relational links to feelings of acceptance and belonging.

Scholars such as Torres (1999, 2003, 2006) have already noted the importance of culture in aged care and have highlighted how understandings of aging are related to cultural values. While it is important to understand these linkages, maintaining links to culture through social networks and connections is also an important source of well-being. The relational aspect of the expression of culture is thus significant because it points to an understanding of culture as more than simply outward engagement in activities and practices, and more than values alone. Rather, culture should be understood as both engagement and embodiment, which is to say that culture informs how people understand who they are and where they belong. As such, culture is something that is both lived and linked to place (Duran & Duran, 1995).

Caidi et al. (2020) referred to the dilemma of belonging when they outlined the experiences of Chinese older adults in Australia, who felt uncertainty about their identity and home, specifically whether they belonged in Australia with their children or with their established networks in China. Rao et al. (2006) noted similar feelings among older adults from CALD backgrounds. Hugman et al. (2004) highlighted that some of the older refugee populations in Sydney felt similarly and expressed a sense of aging in the “wrong place.”

Le et al. (2015) observe that migrants enjoy a positive settlement and integration experience into their host country if the cultural identity of the origin country is maintained and then combined with identification with the host country. Culture, and cultural practice within social networks, thus establishes a connection to the host community that is central to maintaining a sense of identity and belonging for CALD migrant and refugee individuals, and for CALD communities, as they age. Placing culture as central to aging can be the foundation of a broader strategy for older CALD migrants and refugees to reduce isolation and to ensure engagement as equal members of local communities. Anthias (2002, p. 491) argues that useful concepts for examining the processes and outcomes of collective identification are location and positionality (and translocational positionality) because they elucidate how people position themselves with respect to what they do, and do not, belong to.

### Use of Technology for Connection

In order to increase the sense of belonging and reduce isolation for older CALD adults, familiarization with technology and digital information are critical skills for communication and feelings of well-being (Baldassar & Wilding, 2020). Cotten et al. (2013) concluded that the use of the internet is beneficial for older adults as it reduces loneliness, while Lee and Coughlin (2015) identified determinants and barriers to digital literacy among older adults. Shah et al. (2020) referred to the importance of digital technology during pandemic conditions and how an inability to successfully engage with others through technology increased loneliness. The importance of technology to connect with culture and community is a feature demonstrated during periods of COVID-19 lockdown during which people were often isolated and at home.

### Toward a Cultural Well-Being Framework for CALD Migrant and Refugee Aged Care in Australia

Public policies that recognize the shared cultural values of the diverse members of a society, and which strengthen cultural identities in those communities, promote well-being and enhance resilience. They are integral to supporting the social and cultural integration of asylum seekers and refugees (McGregor & Ragab, 2016). Explicit or implicit discrimination and prejudice can undermine the successful integration of asylum seekers and refugees and affect negatively their cultural well-being; for example, when host cultures position migrants as holding views or values that differ from the host culture, which focuses on differences that create feelings of “otherness” (Salma & Salami, 2020). Eliassi (2013, p. 45) notes that “immigrants are constructed as not really belonging to ‘us’, even if they share the same citizenship as ‘us’.” In this paradigm, the well-being of migrants is undermined as modes of belonging, and their presence is often questioned and challenged by majority/dominant members of the host culture.

Many refugees and asylum seekers are exposed to multiple stressors as they move through the migration and resettlement process. Premigration experiences of refugees often include exposure to violence, persecution, vulnerability and loss, etc., while settlement in a host country involves a dynamic interplay of the social, cultural, economic, and political environments in receiving nations that create adverse conditions such as discrimination, marginalization, and inequality that render these groups vulnerable to stress (Choi, 2013; Udah et al., 2019).

In relation to CALD migrants and refugees, culture, cultural practice, and relationship to place are central to creating a new sense of identity and belonging for both individuals and communities as they settle into a host country. The relationship between the “self” and identity is important because “the ‘self’ influences society through actions, and the society influences the self through having shared language and meanings that enable the person to take the role of the other” (Stets & Burke, 2003, p. 128). Identities are constructed and negotiated through a range of interactions, including with institutions, policy, and place, as well as with other groups and individuals.

Migration can provide opportunities for growth and resilience, yet it can also negatively affect well-being. There is a strong relationship between health and well-being and of well-being to economic growth and productivity. Well-being as a field of study has, however, largely been dominated by Psychology, and this has shaped dominant conceptualizations of well-being that emphasize the individual and the individual’s mental health. Such an approach tends to ignore other factors, such as the way in which social, economic, environmental, and cultural policies affect the well-being outcomes of particular social groups (Dalziel et al., 2006). It also neglects the cultural contexts from which we make meaning, including, *inter alia*, food choices and practices, mediation of relationships, and caregiving and receiving in cross-cultural and multicultural contexts (Carlisle & Hanlon, 2008, pp. 264–265; Napier et al., 2014, pp. xi–xii).

A holistic conception of well-being draws upon understandings in relation to Indigenous communities (World Health Organization, 2010), which includes conceptions of culture, strength, self-determination, spiritual links to land, connectedness to ancestors and between individuals, community, the greater universe, and creative practice. Termed “cultural well-being,” this approach is used in relation to public policy for Indigenous peoples by the governments of New Zealand (Reid et al., 2016), as well as in Wales, United Kingdom (CWM TAF Public Services Board, 2018), while a program in Finland from 2010 to 2014 linked culture to health and well-being through art-making (Ministry of Education and Culture, Finland, 2010). For the New Zealand government, this approach focuses on “the vitality that communities and individuals enjoy through participation in recreation, creative and cultural activities [and] the freedom to retain, interpret and express their arts, history, heritage and traditions” (Ministry of Heritage and Culture, New Zealand, 2017). Cultural well-being is thus influenced by the freedom to participate in and practice cultural activities and to belong to a cultural group, but to date its application has been rather limited to Indigenous populations. Indeed, the New Zealand Ministry of Culture and Heritage notes that local councils should feel free to interpret the concept of cultural well-being within the contexts of their own communities (Ministry of Heritage and Culture, New Zealand, 2019). Cultural well-being thus varies during one’s life, including when engaging with settlement in another country.

The application of a cultural well-being framework to aged care for CALD migrant and refugee populations in Australia may serve to increase positive feelings of belonging and connection with community, either coethnic or non-coethnic. While there have not, to our knowledge, been studies of cultural well-being among CALD migrant and refugee aging in other countries, there are certain features of the overall aging experience of migrant populations in the United States and Canada that bear a strong similarity to the Australian experience.

### Comparative International Experience

Understandings of aging are largely culturally determined (Torres, 1999, 2003, 2006). Even if what it means to age is culturally relative, the importance of social networks and connections is clear, especially with close family. One study of perceptions for aging in Latin America and Europe found many older adults, regardless of country of origin or country of resettlement, understood issues around aging in a similar manner, highlighting that being healthy, being able to support themselves, having caring family and friends, and feeling good about themselves are priority attributes (Fernández-Ballesteros et al., 2010).

While the family is important, Acierno et al. (2010) and Bond et al. (2000) noted the dangers of high levels of dependency of older adults with linguistic and cultural literacy limitations in Canada and the United States and warned about the increasing risk of financial, emotional, physical, and sexual abuse by trusted persons. Significantly, while some older adults felt that their families had abused or rejected them, they understood the growing demands on their children and tended to have a more passive approach and disempowered attitude to navigating their new context. Importantly, this finding challenges the assumption that all migrant families “look after their own” (Atwell et al., 2007; Caidi et al., 2020; FECCA, 2015).

The lack of power increased the sense of homesickness and loneliness. This outcome was found to be common among older adults from CALD migrant and refugee backgrounds who lived in countries with multicultural societies. Syed et al. (2017) described the situation among the Chinese-origin older adults in Canada who rarely engaged with local non-coethnics. Kotwal et al. (2021) focused on the impacts of social isolation and poor digital literacy among ethnic older adults in California during the COVID-19 lockdowns. In both cases, language was a major determinant for social isolation, homesickness, and loneliness. The use of technology to promote connectedness and reduce isolation is a feature common to western societies and is quite probably global.

In relation to health care, Ponce et al. (2006) described how language and cultural barriers were major obstacles for older CALD migrants and refugees in California and suggested that their alleviation via appropriate technological tools and personnel training could improve overall health status and services accessibility. While the use of technology can go some way toward reducing social isolation, with respect to health care and settlement services, there is a dimension of language and literacy that affects how well such services can be accessed.

### Limitations

For this study, all articles with a primary focus on medical and/or other chronic conditions were excluded from the review process. Future studies could include such articles so to present a more comprehensive approach regarding the perceptions and needs of a larger older CALD adult population.

## Conclusions

Older CALD adults in Australia felt a sense of disconnectedness from the wider society, especially with respect to feelings of belonging. Low English proficiency was the most common barrier for conditions including loneliness and social isolation. Use of technology and meaningful social contact with family/community networks emerged as key strategies to limited isolation. All the reported barriers affected the capacity of older CALD adults to access various services, especially for health and settlement, which has broader implications for positive health outcomes. Addressing the barriers that exist for older adults requires a nuanced understanding of the intersection of aging, language, sociocultural context, technology, and engagement. Without this understanding, we will not be able to effectively address the barriers that these CALD cohorts face as they age.

At a policy level, it is important to understand the experiences and challenges faced by older adults from CALD migrant and refugee backgrounds in order to meet what is a growing segment of the population. Aging, language, religion/spirituality, sociocultural context, technology, and social engagement are essential components of cultural well-being and should be further investigated. Adopting a cultural well-being framework recognizes that individuals flourish when they are connected to relational and reciprocal family, coethnic and broader community networks. Adopting a framework of cultural well-being in Australia and elsewhere in the world may assist in reducing feelings of isolation, especially among older CALD migrants and refugees, particularly in a post-COVID-19 world.

## Supplementary Material

gnab191_suppl_Supplementary_Material
